# The academic formation challenges: what does retail expect from higher education institutions in pharmacy?

**DOI:** 10.1186/s12909-024-05435-w

**Published:** 2024-04-25

**Authors:** Francielly Lima da Fonseca, Aline Santana Dosea, Fernando de Castro Araújo-Neto, Lívia Gois dos Santos, Déborah Mônica Machado Pimentel, Divaldo Pereira de Lyra

**Affiliations:** 1 Graduate Program in Pharmaceutical Sciences, Laboratory of Teaching and Research in Social Pharmacy (LEPFS), Federal University of Sergipe, São Cristóvão, Sergipe, Brazil; 2 Health Sciences Graduate Program. Laboratory of Teaching and Research in Social Pharmacy (LEPFS), Federal University of Sergipe, São Cristóvão, Sergipe, Brazil; 3 Undergraduate Pharmacy Laboratory of Teaching and Research in Social Pharmacy (LEPFS), Federal University of Sergipe, São Cristóvão, Sergipe, Brazil; 4 Departament of Medicine, Hospital Universitary of Sergipe– Federal University of Sergipe, Aracaju, Sergipe Brazil

**Keywords:** Pharmaceutical retail, Academic education, Qualitative research, Community pharmacy

## Abstract

**Background:**

The drug retail represents the main area of activity for pharmacists worldwide. In Brazil, this sector is responsible for employing around 80% of professionals. Before this reality, the academic training of pharmacists requires specialized skills and knowledge so they can fulfill their tasks. In this sector, considering the influence of managers and mentors on the model of pharmaceutical practice, their perceptions about the demands of the market can help discussions related to the training of pharmacists.

**Aim:**

To analyze the academic training of pharmacists for the drug retail market from the perspective of managers and mentors.

**Method:**

This is a qualitative study conducted with managers and mentors of the drug retail market. A semi-structured interview guide was prepared and applied to the intentionally selected participants. The study was approved by the Research Ethics Committee under the number 4,169,752. The interviews were conducted through videoconference by an experienced researcher. The data obtained were analyzed using Bardin’s analysis technique, following the steps of categorical thematic content analysis using the ATLAS.ti software.

**Results:**

19 interviews were carried out. Among the reports, the interviewees highlighted the importance of retail in the employability of pharmacists, as well as inconsistency in the academic training for this sector, originating the following categories: curriculum reform to include the market demands, follow-up and career plan, training for entrepreneurship and sales, practical application of knowledge, and encouragement of experience.

**Conclusion:**

Pharmaceutical academic training is linked to several challenges, whether organizational, structural, or budgetary. To overcome these challenges, it is necessary to unite the interested parties in the formulation and implementation of a strategy for the professionalization of pharmacists, considering their social role in patient care, aligned with the company’s sustainability, so that both coexist.

**Supplementary Information:**

The online version contains supplementary material available at 10.1186/s12909-024-05435-w.

## Introduction

In recent decades, the insertion of information and communication technologies aligned with globalization has caused changes in the world of labor and proposed new challenges for professional training [1–5]. As a response to the new competitiveness demands that characterize “flexible capitalism”, the world market has required more efficient workers, ready to take risks and open to changes in the search for short-term results [6].

As the knowledge and practice of Pharmacy evolve, significant changes in the profession take place to effectively and safely meet the health needs of the society [7]. Thus, the academic training of pharmacists is changing focus to a greater responsibility on patient care in a more complex and costly health system, breaking with the work model that was historically focused on the production of medicines [8–10]. Therefore, the pharmaceutical training should aim the acquisition of knowledge, skills, and attitudes centered on the work process and decision-making [11–13].

In a worldwide comparison, Brazil has the largest number of higher education courses of Pharmacy (713 courses) [14]. Pharmacists undergo formal training for a period of five years, in addition to participate in continuing education programs provided by professional bodies such as the Federal Pharmacy Council [15]. The Pharmacy Curriculum Guidelines propose competences that must be developed by pharmacists in order to improve their knowledge and develop essential clinical skills for their work, including: communication, leadership, decision making. However, despite realizing the importance of Pharmaceutical Care in promoting the rational use of medicines, there is no standard among Pharmacy courses in Brazil to enable such skills in their curricula [16–19].

According to the Federal Council of Pharmacy (2020), these courses form around 15,000 professionals annually and most of them find the opportunity for their first job in the pharmaceutical retail market [20]. In Brazil, there are more than 90,000 community pharmacies that employ about 81% of professionals, placing Pharmacy in the ranking of professions that employ the most in the country and as the 6th largest market of drug consumption [20].

According to current legislation, community pharmacies in Brazil are now recognized by health systems as an environment that provides care, due to their easy access to the population [21, 22]. In this scenario, the pharmacist assist the population, mostly with vulnerable health and lacking of information, optimizing the effectiveness and safety of the use of medicines and promoting disease prevention [23, 24]. However, this market exposes Brazilian pharmacists to several dilemmas, pressuring them to direct their activities towards business management and the benefit of economic interests [25, 26].

According to the literature, managers and mentors are responsible for making technical decisions and acting in the personal and professional development of pharmacists who work in this area [27–29]. However, few studies consider the influence of these actors on the adaptation of the practice model to the interests of community pharmacies [30–32] and whether their perceptions of the market influence the academic training of pharmacists, helping or not in the understanding of the existence of an alliance between the market aspirations and the social objective of the profession. Therefore, this study aimed to analyze the academic training of pharmacists for the drug retail market from the perspective of managers and mentors.

## Method

### Contextualization

The history of the pharmaceutical profession in Brazil is linked to the arrival of the Portuguese Royal Family in the country, in 1808. During the first three centuries of colonial Brazil, pharmaceutical education was empirically as the university education was prohibited in the domains of the new land33. The first formal Pharmacy courses were established in 1832 in Rio de Janeiro and Bahia, followed by the founding of the first School of Pharmacy in Ouro Preto, Minas Gerais, in 1839 [34, 35].

The regulation of Pharmacy in Brazil only took place in 1960, with the foundation of the Federal Pharmacy Council (CFF), through a federal law. This council is considered the highest instance of the profession in the country and is responsible for regulating and supervising the exercise of the profession, al well, creat guidelines for Pharmaceutical Education, covering since the initial formal training of pharmacists to the continuing education throughout their career [36]. Despite advancements, changes in legislation in 1973 allowed anyone to own a pharmacy, leading to setbacks. However, subsequent events such as the proposal of a National Policy on Access to Medicines, the Brazilian Consensus on Pharmaceutical Assistance and with the creation of guidelines that guide clinical practice helped shape and strengthen the formal training of pharmacists in Brazil [37–41].

Other entities such as the Brazilian Association of Pharmaceutical Education, along with the Pharmacy Course Guidelines, play a crucial role in developing strategies to implement formal education for pharmacists, which typically lasts around five years, with a minimum workload of 4,000 h [42–44]. Despite legislative support for pharmacist autonomy, their role in drugstores is often misunderstood, sometimes leading to subordination to economic interests [44–48].

The Pharmaceutical training in Brazil is generalist, covering research, development, manufacturing, dispensing, and use of medications, as well as other areas like the food industry [36, 49]. During their pharmacy degree, students are encouraged to develop competence for patient care, such as communication [50, 51]. However, the uniformity of this training across the country varies, despite recognizing the importance of pharmaceutical care for rational medicine use [46, 51, 52].

According to the CFF (2023), Brazil has more than 300 thousand registered pharmacists in the country, with 91 thousand of them working in the pharmaceutical retail sector, in community pharmacies or drugstores. Drug dispensing, an exclusive act for pharmacists, is a major service provided, both in public health institutions and private pharmacies [53–56]. While mentoring isn’t mandatory, it’s encouraged by professional organizations to foster personal and professional growth among pharmacists facing the challenges of pharmaceutical practice in Brazil [57–59].

### Study design

This study was carried out between August 2020 and February 2022 and was based on the Consolidated Criteria recommendations for Reporting Qualitative Research, which consists of a 32-item checklist that authors should consider as consolidated criteria for qualitative research reports [60, 61].

A semi-structured interview guide was prepared to explore the perceptions of managers and mentors who work in the drug retail market about the expectations related to the academic training of pharmacists for this practice scenario. To elaborate the script, the authors identified, in the literature, dimensions that were presented as gaps in the understanding of the theme in Brazil, by brainstorming sessions [62–65]. Thus, the research question was: What could you suggest to higher education institutions about the pharmacist academic training?

### Study participants and recruitment

This study was carried out with managers and mentors of the drug retail market who work in large Brazilian pharmacy chains that were in the 2020 Ranking of the *Instituto Brasileiro de Executivos de Varejo & Mercado de Consumo*. According to data from IQVIA (2023), the Brazilian Association of Pharmacy Chains (Abrafarma) holds a significant market share, accounting for almost 11% of the total of 92 thousand pharmacies in the country. Abrafarma employs more than 32 thousand pharmacists and contributes to almost 50% of the revenue of the segment’s in the country (R$ 90 billion). It is estimated that more than 200 thousand people are directly and indirectly impacted by these businesses. In most independent pharmacies in Brazil, the pharmacy owners are laypeople, according to the legislation previously presented, in the contextualization topic. Network pharmacies are where pharmacists are most present. Given the substantial impact of these networks in the country, they were selected to participate in the present study [66, 67].

Initially, all the 11 invited pharmacists agreed to participate in the study. Using the snowball technique, it was possible to recruit more eight participants. This technique consists of a type of non-probability sampling that is used when potential participants are difficult to find or if the sample is limited to a very small subgroup of the population. This technique is quite common in qualitative research and the participant who completes the interview is suggested to nominate another participant to take part in the study [68, 69]. The intention of using this technique was to cover the entire national territory.

At the end of the interviews with all participants, the information collected began to repeat themselves without find new themes, ideas, opinions, or patterns, which is characterized in the literature as data saturation. The proposal of sample saturation was employed as a criterion to finish the inclusion of new participants in the research. As the researchers observed during previous analyses following each interview, recurring themes and redundancy emerged, indicating it was pertinent to conclude the data collection [70]. Moreover, the number of interviews conducted met the necessary average sample size (between 9 and 24 interviews) to achieve data saturation [71].

As this is a qualitative research, the sample is defined by convenience based on the sample saturation criteria [34]. This stratification sought to obtain a differentiated perception of those who have experience related to the community pharmacy market in Brazil. This approach is accepted in qualitative studies because the researcher’s discretion is crucial in determining the relevance of individuals to address the research questions. In this particular study, the aim was to gain in-depth insights into pharmaceutical training within the pharmaceutical retail sector. Therefore, recruiting stakeholders involved the regulation of this sector or those with relevance to pharmacists, who can articulate personal needs for skill enhancement and professional development in response to labor market demands, justifies the intentional sampling. Random sampling, for instance, may not adequately capture such nuanced phenomena at the core of the research inquiry [72–74].

The following characteristics were used as inclusion criteria in both groups of participants:

Group 1- Being a manager or owner of a large Brazilian drugstore or drugstore that was in the IBEVAR 2020 Ranking (Brazilian Institute of Retail & Consumer Market Executives), which orders companies by revenue (IBEVAR, 2020).

Group 2- Being a pharmacist who provides mentoring services to teams at large Brazilian drugstores and/or to individual pharmacists who work in drugstores.

The invitation to the interviewees was carried out by emailing them, with instructions on the purpose of the research, a free and informed consent form to sign, and an online form to collect sociodemographic information via the Google Docs platform. After conducting the initial interviews, new participants were included using the snowball technique in which the interviewee indicates another one subsequently [68, 75].

### Data collection

The interviews were carried out from August to October 2020 remotely, via the Zoom videoconference platform, and were conducted by a researcher trained and experienced in the theme and methodology used, who informed the participants about the conduction of interviews, stimulated spontaneous speeches and was available during the time the interviewees deemed necessary [62]. There were no established relationships between the interviewer and the participants before the study.

No participant refused to participate or abandoned the study. In addition, there was no need to repeat an interview. After performing the interviews, the audiovisual recordings were transcribed and indexed in the ATLAS.ti software for further analysis.

### Data analysis

**Fig. 1:**
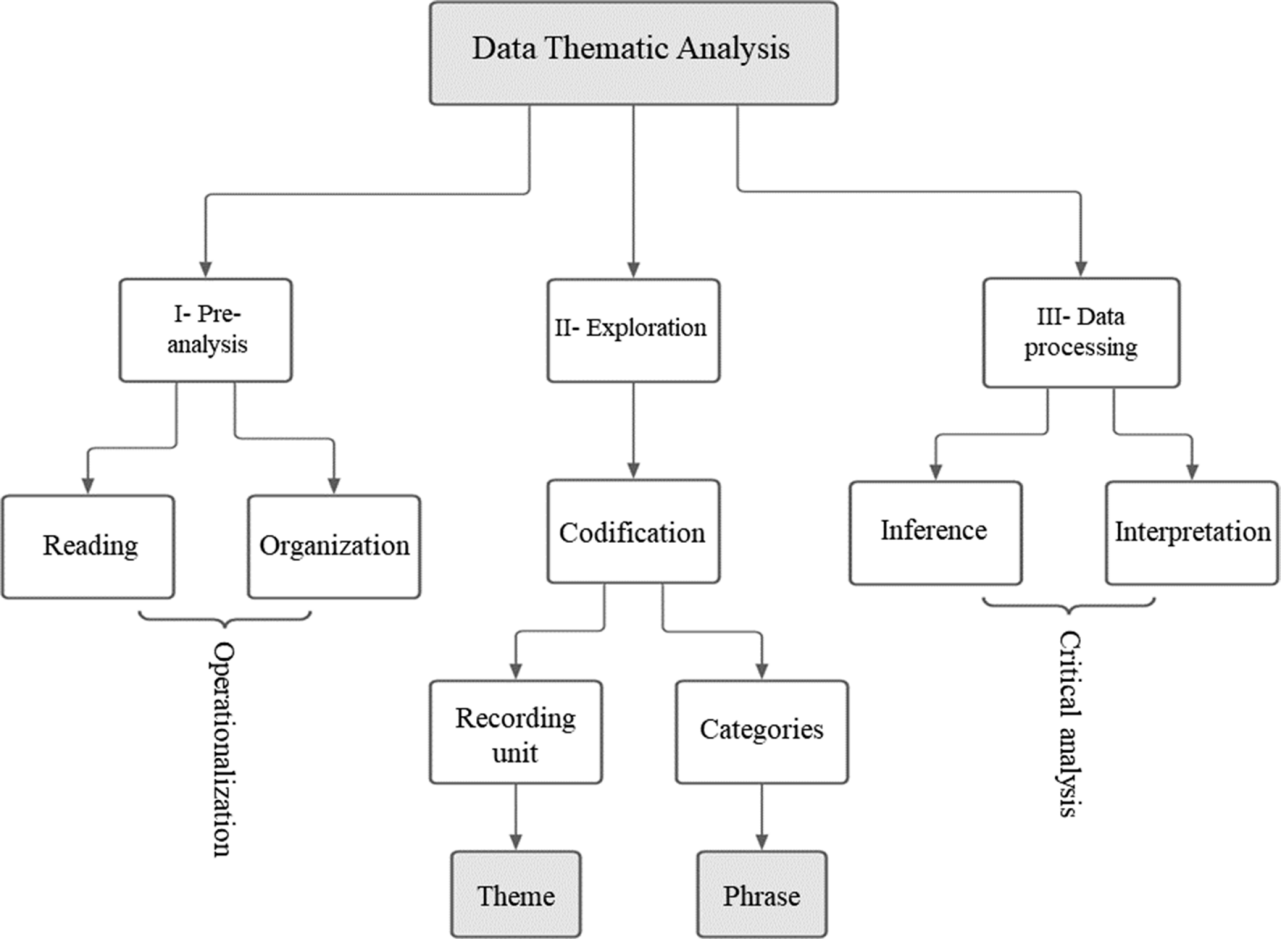
Qualitative data analysis organizational chart. *Source* Prepared by the author, based on Bardin (2016) and Gibbs (2009)

Data were analyzed using the technique of structured data analysis (Content Analysis) proposed by Bardin (2016). Due to the nature of the theme and the familiarity with the method, it was decided to follow the three phases of the thematic categorical content analysis, illustrated in Fig. 1: (a) Pre-analysis; (b) Coding; and (c) Categorization [76]. The pre-analysis and coding stages were carried out in triplicate by three authors of this article (FLF, FCAN, ASD) and the categorization was discussed in brainstorming meetings with the same researchers.

In the pre-analysis, the first material reading was carried out. At this phase, it was possible to identify the first hypotheses of the content analysis process that facilitated the exploration of paths for the content investigation process. In the coding phase, the clippings were selected and transformed into codes related to the research question. The codes emerged inductively from the reports of participants. These reports were documented in the ATLAS.ti software [77]. In the next phase, the semantic categorization was carried out since the adopted registration units were themes. In this phase, the codes were organized and separated by differentiation, grouped by patterns of similarity and causality [78].

Finally, the inferential interpretations in the treatment of the results were based on a reflective and critical analysis based on the theoretical references adopted from the literature about the studied theme. The researchers used quality criteria for the elaboration and consensus of the categories (pertinence, objectivity, homogeneity, mutual exclusion, and productivity) so that they were aligned with the research problem [76, 78]. The study was approved by the Ethics Committee of the Federal University of Sergipe, nº 4.169.752, and the identity of the participants was protected by using the acronyms “G1”; “G2”; “G3” (…) for managers and “M1”; “M2”; “M3” (…) for mentors. During the results, we referenced excerpts from the transcription of the interviews with the same acronyms.

## Results

### Characteristics of the participants

**Fig. 2:**
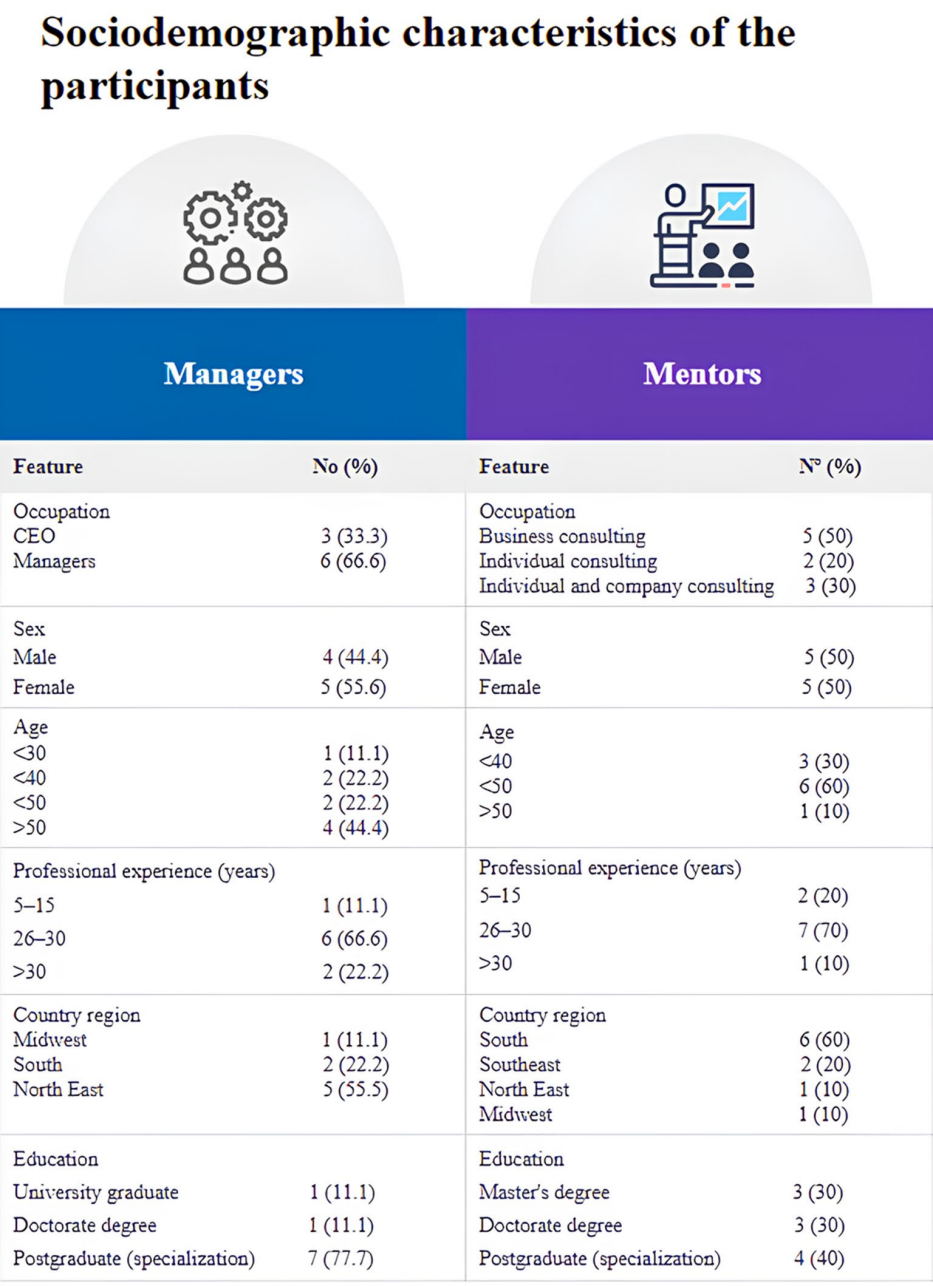
Sociodemografhic characteristics of the participants. *Source* Prepared by the author

Nineteen interviews were carried out, with ten mentors and nine pharmaceutical managers, in an average time of 42 min and a total of 805 min. The sociodemographic characteristics of participants are described in Table 1. Most participants were male (52.6%) aged over 40 years. The time of professional experience varied between six and 32 years, most with more than 15 years of experience. 61% of participants had a *lato sensu* postgraduate degree that was predominantly in Business and Business Management (managers) and Clinical Pharmacy (mentors). The data was summarized in Fig. 2.

**Fig. 3:**
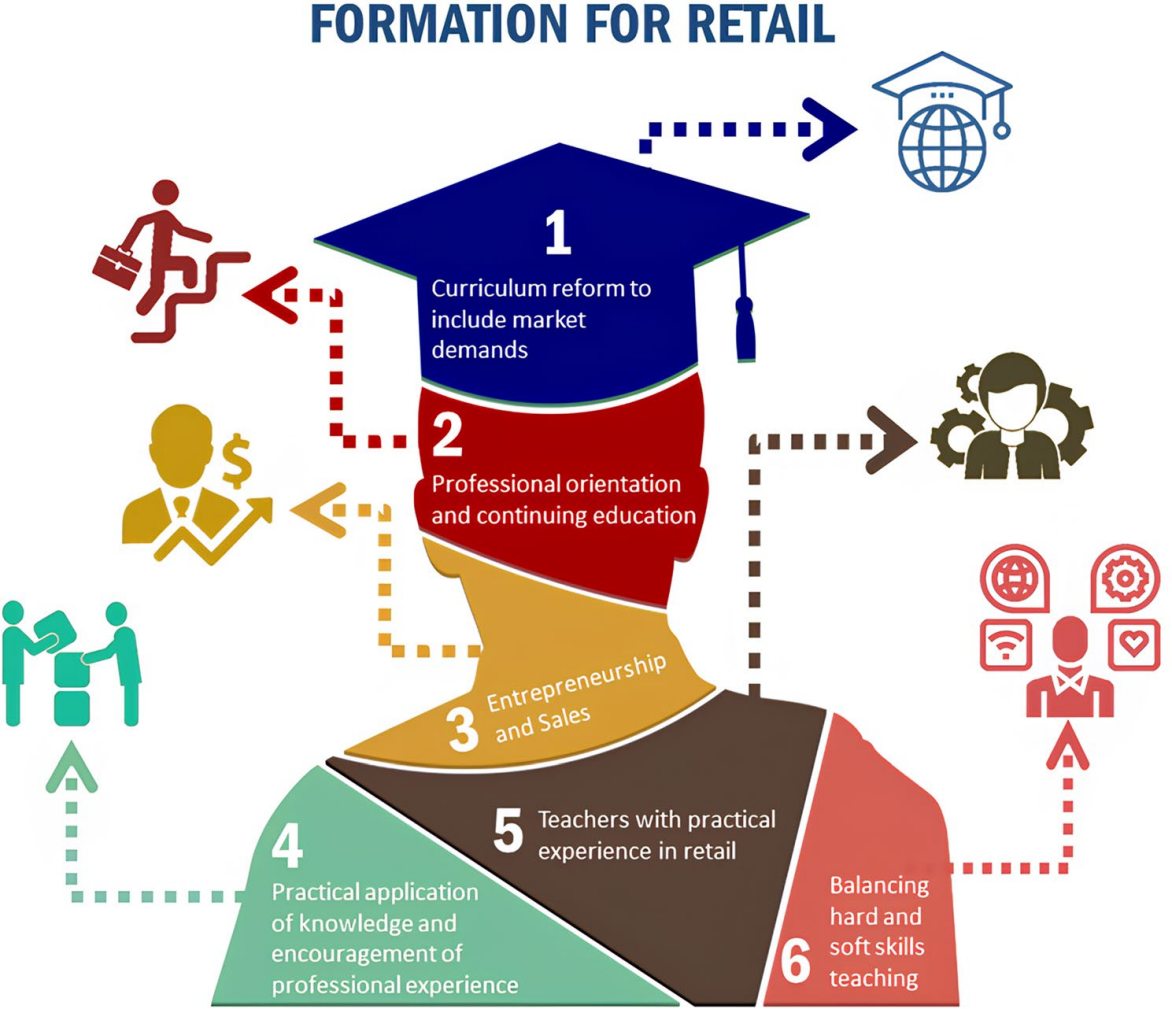
Framework for Pharmacist Formation in Retail. *Source* Prepared by the author

Content analysis pointed to the following categories: curricular reform to include market demands, follow-up and career planning, training for entrepreneurship and sales, practical application of knowledge, encouragement of professional experience (internships, residency, post-graduation), professors with practical experience in retail, and balance between the teaching of Hard and Soft-Skills. These categories are shown in Fig. 3.

### Curriculum reform to include the market demands

In this topic, the interviewees discussed deficiencies in the current Pharmacy curriculum and the difficulty of undergraduate courses in keeping up with the needs of the labor market. In addition, they emphasized that the curriculum is outdated, as it focuses on technical knowledge and basic research that project fictitious scenarios far from reality.

*G1: “Educational institutions do not participate in the market, they are apart, they do not even notice or sometimes look at it with criticism. Not constructive criticism, but like ‘nothing should be done’. A comment that says, efforts are fruitless.”*

*M1: “I think that the Academy is not aware of the real problems and does not direct students to solve the real demands.”*

*G2: “Do I think universities are too utopian? They project reality onto an ideal world that does not exist.”*

The interviewees supported their opinion on the existence of a preconception from universities about the market, due to its commercial and unethical characteristics, which is why universities sometimes see it as an enemy. This causes a reflection that are projected in the curricular disciplines of Pharmacy courses that do not address the fundamental characteristics of working in pharmaceutical retail. Therefore, the interviewees suggest that the curriculum prioritizes aspects such as commodification, management, leadership, and humanization based on the student’s involvement with this reality.

*M2: “Is there a culture in the universities of not selling? You cannot sell. In fact, the sale has to be combined with knowledge.”*

Respondents also highlighted that the retail market is the sector that most employs pharmacists in Brazil. According to their perceptions, more attention should be given to the requirements pointed out by this market for professional training, seeking to achieve desirable levels of quality. Other interviewees, in turn, stated that it is possible to reconcile the curricular bases of Pharmacy courses and the needs of pharmaceutical retail. Among the suggestions there is the inclusion of problem-solving activities associated with the reality of retail in the disciplines. In addition, institutions should approach themselves to retail specialists to solve the shortcomings in the curricula, training professionals to meet the demands of the market.

### Professional guidance and continuing education

Another element highlighted by the interviewees concerns the purpose of the academic training for pharmacists. It was highlighted that Pharmacy students do not receive mentoring from professors during graduation, as it is essential that they are encouraged and guided to the area they identify, feel satisfied, and have the best performance.

*M3: “Where do you go from here? What professional area do you want to specialize in? Do you want to pursue an academic career, as a PhD? So, you want retail, what skills do you have to have then?”*

It was also suggested that even after graduation, pharmacists remain in constant learning and the search for knowledge should guide their personal and professional lives, with continuing education being the means to keep themselves updated.

*M4: “The university will not deliver everything that this professional needs. It is necessary to study constantly and, above all, to have knowledge that is not found in books.”*

### Entrepreneurship and sales training

Teaching focused on entrepreneurship and sales were part of the interviewees’ questions. They emphasized that universities should have disciplines, even if extracurricular, or training that stimulates entrepreneurship, business management, and financial education. Thus, it is believed that pharmacists would have more possibilities for professional growth, leaving the idea of being just an employee and acquiring the mindset of being the owner of their own business.

#### G3

“*First, I think universities could help pharmacists to understand management, leadership, and the administrative area. Why are pharmacists rarely pharmacy owners or entrepreneurs? Why are not they trained for this? The pharmacist is the manager, he is the leader of people, so what I miss most is the administrative part, the business vision, of the profession itself, you know? I think the university fails on that.”*

When questioned about the possibility of charging or not for the services, the informants drew attention to professional valuation, as well as the sustainability of a company from the point of view of sales and profit:

*M5: “Because we have a limiting belief about charging for services, we do not recognize our value, we were not educated to negotiate, sell and value our professional image”.*

*M6: “A health professional sells something– guidance, service– but the pharmacist is not prepared to understand that he is a service provider and he needs to sell himself. Autonomy points out that we need to receive something in return, which is money. This would be the valuation, actualy, but we didn’t have this training.”*

*M7: “We do many things for free, we even give lectures for free. Because we don’t have this training. It’s cultural and we need to break this pattern, because we have personal expenses, our knowledge, our time. So, we need to receive as a form of remuneration, if I deliver something, I should receive something in return, payment, valuation. This is for both service and guidance.”*

On the other hand, some statements exonerate the responsibility of Pharmacy courses by understanding their limitations and the dynamism of knowledge. Therefore, the self-improvement of the pharmacist should be part of its professional routine.

*M8: “In the past, I charged more for universities to train pharmacists for entrepreneurship. Today, I think that this is not a failure of the universities, because even though the curriculum changes with the insertion of management and entrepreneurship disciplines, they do not have enough professors to teach what students need to know to open their pharmacy when they leave the university”.*

### Practical application of knowledge and encouragement of professional experience (internships, residency, postgraduation)

As for the practical application, the importance of associating technical knowledge with practice was reported, as there are differences between the theory that is explored in Pharmacy courses and what is applied in real life. Thus, the interviewees suggested internships, in which students take co-responsibility since the beginning of the course, to gain real experiences that prepare them for the market. In addition, they comment on how residencies and postgraduate courses can be great strategies to complement knowledge and develop practice.

*G4: “When we got to practice, we thought we weren’t prepared. So, internships should be encouraged, favoring contact with trained professionals!”*

*M9: “We have little experience in practical scenarios as students, internships are still precarious. Actualy, we need to delve deeper into the labor market when we leave university, like a residency.”*

### Professors with practical experience in pharmaceutical retail

The importance of professors with practical experience was also discussed. Respondents claim that having this experience influences the student’s development when they go to pharmaceutical retail. In addition, they highlighted the preparedness fragility of some university professors, once having a specialist title is not enough when one does not have professional experience.

*“If you look at the best medical universities, professors act as physicians. They are the best surgeons, cardiologists, etc. who teach. And, in Brazil, how many pharmaceutical professors work in practice? How are you going to form a person if you don’t know the wishes, desires, and needs that pharmacists and patients have?”*

*G6: “It is very different when you have a teacher who has always taught, who has never had practical experience. It can be the best teacher of a certain subject, but it is not equal to working at a pharmacy.”*

### Balance of hard and soft-skills

In this study, the interviewees reported deficiencies in the training of pharmacists regarding their humanization. For this, there must be a balance between the teaching of Hard Skills (technical skills) and Soft skills (socio-emotional skills). Thus, the professional must undergo activities that stimulate emotional work, promoting a better relationship between the pharmacist and the patient, his/her team, and other professionals.

*M8: “To include during training, it doesn’t have to be a curriculum matrix, ok? It can be an extension course or training that offers the development of behavioral skills, emotional intelligence, leadership, and communication. So, what I think is lacking today in institutions is to add this teaching to technical education”*.

*G7: “There must be activities about humanization, and empathy, that encourage the pharmacist to be an available professional, a caregiver.”*

*M9: “Lets work less with technique and more with people’s emotions. Lets balance reason and emotion so that professionals can leverage their future”.*

In Table 1 it is possible to notice differences between the groups of interviewees. Mentors had more comments than managers in all categories except in Practical application of knowledge and encouragement of professional experience. Among the most discussed topics there are Entrepreneurship and sales and Balancing the Hard and Soft Skills teaching. There was an inconsistency among managers and mentors about the Entrepreneurship and sales category, with managers supporting the responsibility of Pharmacy courses for teaching this competence, while mentors believe that it is something very specific and it is up to pharmacists to seek such knowledge. In the other categories, there was agreement between comments.

**Table 1:** Number of times the categories on “Pharmacist education” were addressed among the groups of managers and mentors

Categories	Managers	Mentors	Total
Curriculum reform	18	26	44
Professional guidance and continuing education	10	18	28
Entrepreneurship and sales	25	41	66
Practical application of knowledge and encouragement of professional experience	2	1	3
Teachers without practical experience in retail	4	4	8
Balancing teaching hard and soft-skills	30	32	62
**TOTAL**	**89**	**122**	**211**

*Source* Prepared by the author

## Discussion

As far as we know, this is a first-of-its-kind study that comprehensively investigated the perceptions of pharmacy retail managers and mentors about the academic training of pharmacists for working in community pharmacies in Brazil. The study described the points suggested by the interviewees to overcome the challenges associated with the behavior and professional values of pharmacists pointing out some changes, considering entrepreneurship and technical improvement (Hard-Skills) combined with socio-emotional and humanistic values (Soft-Skills) as essential for success in the labor market.

In recent years, educational policies have been discussed to favor learning by optimizing knowledge, skills, and attitudes necessary for a more qualified performance of pharmacists [79]. In the international literature, it is possible to observe several models and strategies that seek to stimulate the professional development of pharmacists. Most studies reveal the importance of stimulating the communication, empathy, and technical skills of students, as well as the development of critical awareness and professional values and attitudes [80–82].

The negative and positive points detected in this study are aligned with the Pharmacy teaching models implemented in other countries, which corroborates the literature [83–85]. In addition, they are present in the curriculum reforms of Pharmacy courses in developing countries that have similar characteristics to those of Brazil, such as Ghana, Nigeria, Pakistan, and Sri Lanka [86–88]. In 2017, under the influence of the Nanjing conference guide, it is possible to perceive the proposal for changing the Pharmaceutical Education highlighting the need to build an adaptable and capable workforce through transformative and continuous education [89]. Therefore, it is necessary to adopt models of practice, training, and qualification of the Pharmacy teaching institutions for the improvement of students.

From this logic, it was necessary to rethink the administrative, technical, and pedagogical practices adopted by universities, in a critical and committed way, reflecting on their role in society as a producer and socializer of knowledge, capable of understanding and changing the reality [90]. At a national level, the National Guidelines for the Pharmacy course, published in 2017, propose to promote the skills, abilities, and attitudes necessary for meaningful student learning and to bring the pedagogical practice closer to professional reality [91]. However, studies show that most academic training for the pharmacist in Brazil is still centered on the Flexnerian model, focused on the biomedical model, centered on the disease and the hospital. Therefore, students remain with a reductionist view of the health-disease process by excluding the integrality of patient care, reserving little space for social and humanistic outcomes [92–94].

With the new perspective of pharmacist training, the participants of this study highlighted the importance of practice scenarios in improving knowledge. According to Costa, Tonhom, and Fleur (2016), the construction of knowledge must start from the labor market, aiming the praxis through action-reflection-action, to make the practice scenario constant during the teaching-learning process [95]. Thus, the intersection between theory and practice, in an inseparable way, is necessary to transform education, considering the practice scenarios that are the breadbasket for a meaningful and collaborative teaching-learning processes by integrating professors, students, professionals, managers, and patients [96].

The interviewees also reported that the academic experience in practice scenarios is fundamental to the professionalization process of students. According to Melo et al. (2021), controlled and supervised environments are considered essential for professional training, allowing exciting and easy-to-understand experiences for Pharmacy students [97]. It is important to highlight that these scenarios vary in different countries, such as Qatar, United Kingdom, Canada, and Australia, and respect the legal, cultural, religious, and social issues of each location [31, 98–100]. In Brazil, University Pharmacies represent this scenario and are a teaching, research, and extension laboratory aimed at improving pharmaceutical practice, and must be articulated with the political-pedagogical plan of the course [101, 102]. Therefore, the importance of practice scenarios is notable as the key factor to contribute to a positive and effective learning experience, being a useful tool in implementing learning in the workplace and supporting the development of clinical and patient-centered skills.

Despite the advances, the informants of this study pointed out that the training of Brazilian pharmacists is strongly focused on the consolidation of the Brazilian Public Health System, and weakly prone to train these professionals for the demands of the private labor market, which is the main employer in the country [31]. However, it is important to highlight that this data follows the international trend. In the United Kingdom countries, for example, private pharmacies are integrated into the National Health Service (the UK public health system) providing services and pharmaceutical products to the community [103]. This public and private interface allows students to experience different knowledge and apply public health policy programs in different scenarios of professional practice.

In the professional guidance category, the interviewees suggested better reception and direction for undergraduate students. In this sense, the mentoring program can be an alternative, which helps to provide support, knowledge, academic guidance, and professional growth for students [104]. In countries such as Australia, the United States of America, and Belgium, the mentoring model within educational institutions is well established, as professors accompany students during the course, providing emotional and academic support both in teaching and internship spaces [104–106]. In Brazil, the implementation of mentoring programs is still scarce [107]. This is due, in great part, to the unavailability of professors to participate as mentors, which may be linked to the reduced number of professionals that composes the academic chair, leaving them overloaded due to the amount of designated assignments [108].

On the other hand, mentoring in the pharmaceutical labor market has become a very common tool in the development of continuing education. Studies indicate that implementing mentoring programs in pharmaceutical organizations and professional bodies improves professional guidance and contributes to a learning culture within the pharmacy [109, 110]. However, several factors interfere when choosing a mentoring model to be implemented, being necessary to reflect on the critical points to be solved and consider how each factor can be contextualized to support them in achieving their overarching objective. Another important resource in continuing education is the residency in Pharmacy. It is configured as an opportunity in the process of acquiring leadership training and knowledge production allied with the experience of real professional practices that allow the deepening of knowledge and development of skills and attitudes that raise the specialization degree [111–113].

Informants agreed that pharmaceutical services are essential in maintaining and recovering the health of patient and, when monetized, the needs of patients remain prioritized, making it possible to profit from products and services in an ethical, responsible, and transparent manner [26, 114]. On the other hand, the evaluation of the productivity of pharmacists remains based on goals and indicators that prioritize the commercial interests of pharmacy chains, such as sale of products and services, leaving the patient health needs in the background [103, 115, 116]. This means that changing the way of promoting health care challenges the sustainability of these systems. But the remain question is how much pharmacy chains are willing to invest on health services to the detriment of profit focused on the product, once, economically speaking, the predominant business model is still one of the strongest in the world [117, 118].

The interviews revealed that the academic and research field suffer constant pressure from society about the formation of professionals for the labor market, as the graduates must be qualified to enter in the production system. In this sense, professors are deemed to have professional experience in the labor market [119]. Although knowledge about a specific professional field is an inseparable value of its training, ethical values remove the emphasis given to a training model that leads only to technical super-specialization, under the market pressure [120]. Therefore, Freidson (2009) points out the need of teachers distancing themselves from the labor market [121]. The author justifies that since the faculty relies on an academic and non-commercial market, it is isolated from the practical demands of the everyday world. Therefore, an ethical-political education should prioritize technical-scientific qualifications, moving away from mercantilist proposals that often mischaracterize the role of universities.

In the results, entrepreneurship appears as a key element for the pharmacist to stand out in the labor market, perceived as a topic of great relevance in the business world. Internationally, most pharmacy owners are pharmacists. This is mainly due to a perspective of seeing the pharmacy as a health environment and the importance of the pharmacist in the management and safety of patients in the use of medicines [122, 123]. In the national context, according to research carried out by the Global Entrepreneurship Monitor (2022), three out of ten Brazilian adults aged between 18 and 64 own a company or are involved in creating their own business [124]. This happens because many people see entrepreneurship as a life opportunity to own their own business. On the other hand, these data do not reflect the reality of pharmacists, in which only 16% are entrepreneurs and more and more independent pharmacies close their doors [125, 126]. This may be linked to the lack of tax incentives and high taxes that increase the indebtedness of the small entrepreneurs, as well as the unfair competition with the large conglomerates of pharmacy chains.

Another suggestion highlighted in the interviews to improve pharmacist training given the needs of the drug retail market was to associate the teaching of Hard-Skills and Soft-Skills in the curriculum of undergraduate Pharmacy students. This demand occurs due to the new market demands for these skills [127, 128]. Thus, Schulz (2008) presents strategies to insert them into the higher education curriculum, among which there is the formal training. That is why Pharmacy students must participate in disciplines, lectures, and courses that teach technical and practical knowledge with a central focus on the development of soft skills, intensifying student engagement [128, 129].

The findings of this study corroborate the international literature by identifying deficiencies in the training of pharmacists and its incompatibility with reality. This lack of relevance seems to be closely related to the skills that pharmacists need to develop to provide quality health services. Although the interviewees outlined significant competencies for pharmacist training, there is no consensus on how they can be achieved. Incorporating these skills for a better performance of the pharmacist in the labor market is fundamental for more patient-focused conducts. However, it is a challenge, since the international competition among companies, the pressure to minimize the cost of work, and the globalization of markets and capital, have caused drastic changes in the labor market, giving rise to a flexible and uberized workforce. This flexibility has caused a vulnerability in the performance of pharmacists, a significantly growth of the precariousness of their work, the displacement of attributions and low remuneration. Finally, in Brazil, the abandonment of functions that are inherent to retail pharmacists cannot be exclusively attributed to the academic training but is closely related to government policies and market interests.

Yet, it is necessary to consider that pharmaceutical education is not linear, and its history has been through numerous proposals to change and standardizing the curricula, influencing the formation, reform, or deformation of the social role of the pharmacist. Currently, the pharmaceutical profession is inserted in a market that often disregards the needs of patients to favors profit. Despite the considerations in establishing the retail pharmacy as a health environment, market competitiveness and economic interests put pressure on pharmacists who experience conflicts and ethical dilemmas in the duality of “commerce” versus “health”. Therefore, pharmacists need to understand that their social role in patient care must be aligned with the sustainability of the company so that both coexist.

## Limitations

A potential limitation of our study is the lack of a set of other stakeholders (including patients, pharmacists, and other pharmacy staff). This study involved samples of pharmacists from different regions of Brazil, a country with continental characteristics. Thus, although the sample was diverse with different characteristics and realities, the representativeness of the broader population of pharmacists in any country cannot be assumed, considering the political and organizational peculiarities of pharmaceutical practice. However, the data revealed results that agree with the existing literature and therefore can be expected to resonate with pharmacists beyond the sample. The structure and operations of community pharmacies in many parts of the world have many common features: they generally operate in the private sector, with specific responsibilities in primary care. Future research involving a wider range of participants and that include other scenarios, such as hospital pharmacy or personal pharmacy would improve and confirm our findings.

## Conclusion

The present study allows inferring some reflections related to the organizational, structural, and budgetary challenges that can support the change of the academic training profile of pharmacists prepared and dedicated to meet the demands of society. For this, it is necessary that teachers are committed to obtain new skills that optimize both the students’ and their own learning. Furthermore, they need to have the attitude to participate in the process of construction, implantation, and implementation of curricular innovations to break with the traditional model.

### Electronic supplementary material

Below is the link to the electronic supplementary material.

Supplementary Material 1

## Data Availability

The datasets used and/or analysed during the current study are available from. the corresponding author on reasonable request.
