# Supplementary material for: The academic formation challenges: what does retail expect from higher education institutions in pharmacy?
Source: BMC Med Educ. 2024 Apr 25;24:456. doi: 10.1186/s12909-024-05435-w (PMC11046822; doi:10.1186/s12909-024-05435-w)
Supplement: Supplementary file 1 — Supplementary Material 1 [file 12909_2024_5435_MOESM1_ESM.docx]

**SUPPLEMENTARY MATERIAL**

Complete script of the interviews presented in the master's thesis

*1- Thinking about the pharmaceutical professional, what is the best definition of professionalism?*

*2- What would an ideal pharmacist be like for you (thinking about the job market/pharmaceutical retail)?*

*3 - How can pharmaceutical services add value to your company/pharmaceutical retail?/how can pharmacists value their work in retail?*

*4- How do you think pharmacist autonomy is and should be in the retail drug market setting?*

*5- What can be done to improve pharmacists’ professional autonomy?*

*6- How are the ethical dilemmas faced by pharmaceutical retailers interpreted in Brazilian community pharmacies?/How do you believe the ethical limits of the relationship between the industry and the pharmacist should be?*

*7- In the second question, you described the ideal pharmacist, thinking about what these pharmacists lack to get closer to what you described... What can you suggest to higher education institutions about pharmacist training?*
